# Early detection of COVID-19 in the UK using self-reported symptoms: a large-scale, prospective, epidemiological surveillance study

**DOI:** 10.1016/S2589-7500(21)00131-X

**Published:** 2021-07-29

**Authors:** Liane S Canas, Carole H Sudre, Joan Capdevila Pujol, Lorenzo Polidori, Benjamin Murray, Erika Molteni, Mark S Graham, Kerstin Klaser, Michela Antonelli, Sarah Berry, Richard Davies, Long H Nguyen, David A Drew, Jonathan Wolf, Andrew T Chan, Tim Spector, Claire J Steves, Sebastien Ourselin, Marc Modat

**Affiliations:** a School of Biomedical Engineering and Imaging Sciences, King's College London, London, UK; b Department of Twin Research and Genetic Epidemiology, King's College London, London, UK; c Medical Research Council Unit for Lifelong Health and Ageing, Department of Population Science and Experimental Medicine, University College London, London, UK; d Centre for Medical Image Computing, Department of Computer Science, University College London, London, UK; e ZOE, London, UK; f Clinical and Translational Epidemiology Unit, Massachusetts General Hospital and Harvard Medical School, Boston, MA, USA

## Abstract

**Background:**

Self-reported symptoms during the COVID-19 pandemic have been used to train artificial intelligence models to identify possible infection foci. To date, these models have only considered the culmination or peak of symptoms, which is not suitable for the early detection of infection. We aimed to estimate the probability of an individual being infected with SARS-CoV-2 on the basis of early self-reported symptoms to enable timely self-isolation and urgent testing.

**Methods:**

In this large-scale, prospective, epidemiological surveillance study, we used prospective, observational, longitudinal, self-reported data from participants in the UK on 19 symptoms over 3 days after symptoms onset and COVID-19 PCR test results extracted from the COVID-19 Symptom Study mobile phone app. We divided the study population into a training set (those who reported symptoms between April 29, 2020, and Oct 15, 2020) and a test set (those who reported symptoms between Oct 16, 2020, and Nov 30, 2020), and used three models to analyse the self-reported symptoms: the UK's National Health Service (NHS) algorithm, logistic regression, and the hierarchical Gaussian process model we designed to account for several important variables (eg, specific COVID-19 symptoms, comorbidities, and clinical information). Model performance to predict COVID-19 positivity was compared in terms of sensitivity, specificity, and area under the receiver operating characteristic curve (AUC) in the test set. For the hierarchical Gaussian process model, we also evaluated the relevance of symptoms in the early detection of COVID-19 in population subgroups stratified according to occupation, sex, age, and body-mass index.

**Findings:**

The training set comprised 182 991 participants and the test set comprised 15 049 participants. When trained on 3 days of self-reported symptoms, the hierarchical Gaussian process model had a higher prediction AUC (0·80 [95% CI 0·80–0·81]) than did the logistic regression model (0·74 [0·74–0·75]) and the NHS algorithm (0·67 [0·67–0·67]). AUCs for all models increased with the number of days of self-reported symptoms, but were still high for the hierarchical Gaussian process model at day 1 (0·73 [95% CI 0·73–0·74]) and day 2 (0·79 [0·78–0·79]). At day 3, the hierarchical Gaussian process model also had a significantly higher sensitivity, but a non-statistically lower specificity, than did the two other models. The hierarchical Gaussian process model also identified different sets of relevant features to detect COVID-19 between younger and older subgroups, and between health-care workers and non-health-care workers. When used during different pandemic periods, the model was robust to changes in populations.

**Interpretation:**

Early detection of SARS-CoV-2 infection is feasible with our model. Such early detection is crucial to contain the spread of COVID-19 and efficiently allocate medical resources.

**Funding:**

ZOE, the UK Government Department of Health and Social Care, the Wellcome Trust, the UK Engineering and Physical Sciences Research Council, the UK National Institute for Health Research, the UK Medical Research Council, the British Heart Foundation, the Alzheimer's Society, the Chronic Disease Research Foundation, and the Massachusetts Consortium on Pathogen Readiness.

## Introduction

COVID-19 is an acute respiratory illness caused by SARS-CoV-2.^1^ Between Dec 1, 2019, and June 10, 2021, this illness affected more than 175 million individuals worldwide, according to Worldometer. By Jan 31, 2021, the UK alone had recorded 38 163 patients requiring hospitalisation for COVID-19 (Our World in Data). In such circumstances, health-care infrastructures suffer extraordinary and overwhelming demand for resources and require fast, drastic rationing, which can easily result in poor outcomes (eg, long-term morbidity and death).^2^ The efficient allocation of resources is essential to manage the pandemic's long-term effects, not only for the treatment of patients with COVID-19, but also for those with other critical medical conditions. At the time of writing, health-care infrastructures (eg, the UK's National Health Service [NHS]) are facing enormous pressure in managing clinical resources as a high number of patients are in critical condition and require special care.^2^ This pressure could be reduced if the infection rate and the number of severe cases are decreased. One way to decrease the infection rate is via the timely detection of patients with COVID-19, through which the spread of disease can be contained while the evolution of symptoms can be treated.^3^, ^4^, ^5^ However, the widespread testing of the population and the identification of patients positive for COVID-19 are still difficult; laboratory professionals have faced a growing list of challenges as they have attempted to balance the need for increased test capacity with the maintenance of high-quality laboratory operations.^4^ Furthermore, approaches used for diagnosing current infection, such as PCR, are highly dependent on the timing of sample collection and the type of sample acquired. Indeed, SARS-CoV-2 might not be detected by conventional PCR tests using samples obtained via upper respiratory tract swabs because viral presence in the upper respiratory tract decreases during the first week after symptom onset.^4^ Thus, as a complementary approach, identifying the combination of symptoms that enables early prediction of COVID-19 is essential. Such prediction could help to promote the timely testing of people with suspected SARS-CoV-2 infection.^6^, ^7^, ^8^, ^9^

**Research in context**

**Evidence before this study**

Given the growth of surveillance platforms to investigate signs of SARS-CoV-2 infection and the progression of COVID-19, we designed a study to examine the early detection of this illness. We searched PubMed for peer-reviewed articles published in any language between Jan 1, 2020, and Jan 1, 2021, using the keywords “COVID-19” AND ([“mobile application”] OR [“web tool”] OR [“digital survey”] OR [“early detection”]). Of the 446 results, we found nine studies that used self-reported symptoms to predict signs of SARS-CoV-2 infection. Among them, one study showed positive results in detecting COVID-19 in a general population on a national level. However, none of the studies showed evidence of the early detection (eg, in the first 3 days) of COVID-19; most focused on the detection of COVID-19 during the peak of symptoms. Furthermore, none of the studies sought to provide comparisons with current diagnostic criteria used by health-care services, showing the added value of artificial intelligence technologies to model the early signs of the disease explicitly.

**Added value of this study**

This study represents a novel effort to identify and refer individuals for COVID-19 testing, enabling a more efficient allocation of medical resources during crucial stages of a pandemic. Our results suggest that a hierarchical Gaussian process model is effective in predicting SARS-CoV-2 infection when earlier symptoms are considered. Furthermore, this study used a uniquely large dataset comprising a prospective, community-based cohort. The longitudinal information provided by the participants also allowed the temporal assessment of the different symptoms collected.

**Implications of all the available evidence**

Our findings show the value of artificial intelligence in modelling COVID-19 symptoms and in the timely detection of SARS-CoV-2 infections. Such a model would enable the prompt self-isolation of individuals with suspected COVID-19 and referral for urgent testing, enabling better allocation of medical resources during an evolving pandemic, particularly during chaotic periods.

The COVID-19 Symptom Study app^6^ collects self-reported, longitudinal information about the symptom profiles of patients with COVID-19 (confirmed by a PCR test). Machine learning approaches have been developed by use of the information collected from the COVID-19 Symptom Study app^6^ to create diagnostic models and leverage the added value of big data to study new diseases such as COVID-19.^7^, ^10^ Despite some promising results in identifying the main symptoms of COVID-19 and their correlation to outcomes, most proposed models only use the information available at the time of maximum symptom intensity (defined as the peak of symptoms), and, hence, are not as conducive for early diagnosis.

To identify who is most likely to have COVID-19 on the basis of early symptoms, we aimed to create a Bayesian model that uses the 3 initial days of self-reported symptoms collected via the COVID-19 Symptom Study app.^6^ When optimised, the model will assign each participant who reports their daily symptoms with a likelihood of being COVID-19-positive. This information could then be used as a proxy for clinical diagnosis, signalising the individual for either a PCR test or self-isolation while waiting for SARS-CoV-2 test results.

## Methods

### Data sources

The data used in this study are part of a prospective dataset acquired by use of the COVID-19 Symptom Study app: a mobile health application developed by ZOE (London, UK) in collaboration with scientists from King's College London (London, UK) and Massachusetts General Hospital (Boston, MA, USA).^6^ The data source and study design have been previously described in detail in a validation study, which provided the basis for ethical approval of this study. Briefly, the COVID-19 Symptom Study app collected self-reported data on 19 symptoms and COVID-19 PCR test results (appendix p 1). For the purposes of this research, we used 18 of these self-reported symptoms, excluding red welts on the face and lips due to possible confounding assessments for this symptom, and the study population was limited to participants in the UK. The app and the COVID-19 Symptom Study were approved by King's College London's ethics committee (Research Ethics Management Application System number 18210; review reference LRS-19/20–18210) and all app users provided informed digital consent for use of their data in COVID-19 research.

From the data acquired by the mobile app, we extracted a subset of longitudinal information for 3 days of self-reported symptoms after symptom onset. Symptoms encoding and description are available in the appendix (pp 1–2). All samples with invalid data entries or incomplete information were excluded from this study. The resulting dataset included a total of 182 991 samples (participants) who reported symptoms between April 29, 2020, and Oct 15, 2020 (the training set), and an additional population comprising 15 049 participants who recorded their symptoms between Oct 16, 2020, and Nov 30, 2020 (the test set). All participants were labelled according to their self-reported PCR test results as being either positive or negative for SARS-CoV-2 infection, which was then used as the diagnostic criteria for both model training and evaluation. To compensate for dataset imbalance in the number of positive versus negative cases during training, we implemented a bootstrapping scheme to increase the proportion of SARS-CoV-2-positive participants in the training set from the initial 2% to 5%. We used five bootstrapping folds on the training set to train the models in a subsample of 80% of the set; the remaining 20% were used for testing.

### Data analysis and models definitions

We used three approaches to analyse the self-reported symptoms. The three approaches differ according to the number of symptoms considered as relevant to diagnose COVID-19 and in their temporal encoding. The first method mimics the standard approach implemented by the UK's NHS for triage and test referencing.^11^ This method, hereafter referred to as the NHS algorithm, considers all individuals reporting either cough, fever, or loss of smell in any of the days in a time window (varying from 1 day to 3 days in our study) as potentially being infected with SARS-CoV-2.

The second approach identifies patients with COVID-19 on the basis of a symptomatic profile accumulated over time of a subset of symptoms (ie, loss of smell, persistent cough, fatigue, and skipped meals), which were previously validated to detect COVID-19,^7^ and demographic information by use of a logistic regression model proposed by Menni and colleagues.^7^ Further details regarding model definitions and implementation are available in the appendix (p 5).

For the third approach, we designed a Bayesian framework to model the relationship between self-reported symptoms, comorbidities (eg, diabetes, kidney disease, heart disease, and lung disease), and an individual's COVID-19 status. 18 self-reported symptoms were used by this model. We also included demographic information as covariates in the model, namely age, sex, body-mass index (BMI), occupation according to SARS-CoV-2 exposure (health-care workers *vs* non-health-care workers), and the risk of contact with infected patients for health-care workers during the COVID-19 pandemic; further details can be found in the appendix (pp 5–6). Bayesian frameworks, such as the Gaussian process, are particularly useful to study SARS-CoV-2 infection, as they allow robust modelling even in highly uncertain or incomplete datasets, such as medical datasets.^12^ Unlike the previous two models, the proposed model, defined as a hierarchical Gaussian process model (appendix pp 5–6), encodes both the correlation between symptoms across participants and the correlation between timepoints (temporal evolutions), considering the prevalence ratio of COVID-19 associated with the geographical area where participants reside.^13^ Furthermore, our hierarchical Gaussian process model encodes the ordering of symptoms as a time-series sequence across the different participants, and within participants across timepoints (appendix pp 5–6).

The hierarchical Gaussian process model was optimised by use of the open-source software GPflow, version 2.2,^14^ and TensorFlow, version 2.0. Both the logistic regression model and the NHS algorithm were developed by use of Python, version 3.7. The study design and data are registered in ClinicalTrials.gov, NCT04331509.

### Power of the models to predict COVID-19 positivity

The prediction of SARS-CoV-2 infection was obtained independently for the three models considered. The NHS algorithm and the logistic regression models were evaluated by use of the sum of the maximum values of the symptoms reported by participants across time for the test population (most were binary encoded; appendix p 1). Conversely, the results for the hierarchical Gaussian process model were computed independently for each timepoint. All models were compared in terms of sensitivity, specificity, and area under the receiver operating characteristic curve (AUC) in the test set. Three thresholds were considered to define positive cases from the likelihood of predictions to maximise the sensitivity (denoted as high sensitivity), specificity (denoted as high specificity), and optimal threshold (which balances sensitivity and specificity), defined by Youden's J statistic.^15^ The optimisation of predictive thresholds was done on the training set and subsequently applied to the testing set.

The differences in performance across the three models were assessed by use of a Mann–Whitney *U* test, with significance levels of 95% and 99% (not reported). A multiple comparison correction was done for the 3 days of self-reported symptoms.

We further planned to analyse the sensitivity, specificity, and AUC (using the optimal threshold) of the best performing model when trained in subgroups of the population stratified according to occupation (health-care worker *vs* non-health-care worker), sex (female *vs* male), age (16–39 years *vs* 40–59 years *vs* 60–79 years *vs* ≥80 years), and BMI (underweight [<18·5 kg/m^2^] *vs* healthy weight [18·5–24·9 kg/m^2^] *vs* overweight [25·0–29·9 kg/m^2^] *vs* obese [≥30·0 kg/m^2^]).

### Stratification of feature relevance in population subgroups

Feature (symptom) relevance was extracted from the optimised hierarchical Gaussian process model as a surrogate metric to identify the most relevant symptoms indicating early signs of COVID-19 in different population subgroups and to assess the relevance to COVID-19 status prediction of self-reported comorbidities. As defined in the clinical trial experimental setup, we considered the same four subgroups as in our performance analysis, stratified according to occupation, sex, age, and BMI. To more easily analyse the relevance of different symptoms, we grouped them into four groups according to their clinical manifestations: (1) gastrointestinal symptoms and other symptoms; (2) flu-like symptoms; (3) neurological symptoms; and (4) cardiac and respiratory symptoms (appendix p 1). This symptom grouping was a post-processing step that facilitated results interpretation, and did not influence model performance. Stratification of feature relevance was obtained by optimising the prediction model in these subgroups of the population (appendix pp 3–4). We used a Kruskal–Wallis test with Bonferroni correction to assess statistical differences in the distribution of symptom relevance across population groups. The weights for each feature were normalised between 0 and 1 using the maximum value of the weights of the features across the subgroups.

### Confidence of label prediction

Given the Bayesian nature of the hierarchical Gaussian process model, we further analysed the uncertainty of the predicted labels (ie, positive or negative for SARS-CoV-2 infection) using the likelihood of the correctly labelled classes to understand how confident the model was in predicting COVID-19 positivity among age, sex, occupation, and BMI subgroups (appendix p 9).

### Role of the funding source

The funders of the study had no role in study design, data analysis, data interpretation, or writing of the report. ZOE participated in data acquisition and no other funders participated in data collection.

## Results

We included 182 991 participants in the training set and 15 049 participants in the test set. The two populations had similar symptom distributions (appendix p 2) and demographics (table 1), and were completely independent. A detailed description of the population stratified into age and BMI subgroups is available in the appendix (pp 3–4).

**Table 1:** Demographic information of the study population

		**SARS-CoV-2-positive**			**SARS-CoV-2-negative**		
		1 day	2 days	3 days	1 day	2 days	3 days
Number*							
Training set		1965 (1·3%)	1057 (1·7%)	997 (1·9%)	144 490 (98·7%)	60 114 (98·3%)	52 532 (98·1%)
Testing set		1158 (7·7%)	752 (11·1%)	679 (13·3%)	13 891 (92·3%)	5993 (88·9%)	4439 (86·7%)
**Sex**†							
Male							
	Training set	537 (27·3%)	276 (26·1%)	262 (26·3%)	36 601 (25·3%)	13 889 (23·1%)	11 901 (22·7%)
	Testing set	334 (28·8%)	211 (28·1%)	193 (28·4%)	3422 (24·6%)	1342 (22·4%)	1001 (22·6%)
Female							
	Training set	1428 (72·7%)	781 (73·9%)	735 (73·7%)	107 889 (74·7%)	46 225 (76·9%)	40 631 (77·3%)
	Testing set	824 (71·2%)	541 (71·9%)	486 (71·6%)	10 469 (75·4%)	4651 (77·6%)	3438 (77·4%)
**Age, years**							
Training set		46·7 (14·3)	46·9 (14·3)	46·5 (14·2)	49·3 (13·2)	49·4 (13·0)	49·6 (12·8)
Testing set		50·3 (12·7)	50·0 (12·5)	50·0 (12·6)	50·8 (12·8)	51·2 (12·6)	51·2 (12·5)
**BMI, kg/m^2^**							
Training set		27·4 (6·9)	27·4 (6·8)	27·4 (6·8)	27·2 (7·0)	27·1 (6·9)	27·2 (6·9)
Testing set		27·8 (7·0)	27·9 (7·2)	27·7 (7·1)	27·1 (6·9)	27·0 (6·8)	27·1 (6·9)
**Health-care workers**†							
Training set		189 (9·6%)	125 (11·8%)	113 (11·3%)	7045 (4·9%)	2985 (5·0%)	2463 (4·7%)
Testing set		48 (4·1%)	32 (4·3%)	30 (4·4%)	649 (4·7%)	266 (4·4%)	179 (4·0%)

Data are n (%) or mean (SD). Data are stratified by the number of days after symptom onset. BMI=body-mass index.

* Denominators are the total number of participants in each set for each day.

† Denominators are the training or testing set numbers of participants who are either positive or negative for SARS-CoV-2 each individual day (the first two rows).

The ability to predict the COVID-19 test result after a maximum of 3 days of self-reported symptoms was assessed via the sensitivity, specificity, and AUC of the three models (table 2). The model was trained with data from April 29, 2020, to Oct 15, 2020, and tested with data from Oct 16, 2020, to Nov 30, 2020, and appeared robust to changes in the underlying pandemic periods (table 2). The hierarchical Gaussian process model showed the highest prediction AUC, which increased with the number of days of self-reported symptoms (0·73 [95% CI 0·73–0·74] for 1 day, 0·79 [0·78–0·79] for 2 days, and 0·80 [0·80–0·81] for 3 days; appendix p 7). These results suggest that the model performed better when using information about the temporal evolution of symptoms. Nevertheless, sensitivity was similar across the three timepoints for the hierarchical Gaussian process model, whereas specificity benefited from the temporal information, increasing with the number of days of self-reported symptoms (table 2).

**Table 2:** Overall performance metrics in the test set

	**Sensitivity**			**Specificity**			**AUC**
	High sensitivity	High specificity	Optimal threshold	High sensitivity	High specificity	Optimal threshold	
**1 day**							
Logistic regression	0·87 (0·02; 0·85–0·89)*	0·29 (0·02; 0·27–0·30)*	0·43 (0·06; 0·40–0·49)*	0·22 (0·03; 0·19–0·24)	0·89 (<0·01; 0·89–0·89)*	0·77 (0·05; 0·73–0·81)*	0·64 (0·01; 0·63–0·65)*
Hierarchical Gaussian process	0·95 (0·01; 0·94–0·95)*	0·49 (0·04; 0·46–0·53)*	0·76 (0·06; 0·71–0·80)*	0·16 (0·02; 0·15–0·20)	0·83 (0·02; 0·81–0·85)*	0·57 (0·08; 0·51–0·64)*	0·73 (<0·01; 0·73–0·74)*
**2 days**							
Logistic regression	0·91 (0·01; 0·90–0·92)*	0·34 (0·01; 0·33–0·35)*	0·58 (0·06; 0·52–0·63)*	0·24 (0·03; 0·21–0·27)	0·90 (0·01; 0·90–0·91)*	0·73 (0·06; 0·68–0·79)	0·71 (0·01; 0·70–0·71)*
Hierarchical Gaussian process	0·94 (0·01; 0·93–0·95)*	0·57 (0·04; 0·54–0·59)*	0·75 (0·04; 0·72–0·78)*	0·29 (0·03; 0·27–0·31)	0·84 (0·01; 0·83–0·86)*	0·68 (0·04; 0·64–0·71)	0·79 (<0·01; 0·78–0·79)*
**3 days**							
NHS algorithm	..	..	0·60 (0·02; 0·58–0·62)†‡	..	..	0·75 (<0·01; 0·75–0·75)	0·67 (<0·01; 0·67–0·67)†§
Logistic regression	0·91 (0·03; 0·88–0·94)	0·36 (0·02; 0·34–0·37)†	0·59 (0·06; 0·54–0·65)†	0·31 (0·04; 0·26–0·33)	0·91 (0·01; 0·90–0·91)†	0·76 (0·06; 0·71–0·81)	0·74 (0·01; 0·74–0·75)†
Hierarchical Gaussian process	0·95 (0·01; 0·93–0·95)	0·59 (0·03; 0·57–0·61)†	0·73 (0·05; 0·69–0·77)†	0·31 (0·04; 0·28–0·35)	0·85 (0·01; 0·84–0·86)†	0·72 (0·02; 0·70–0·73)	0·80 (<0·01; 0·80–0·81)†

Data are mean (SD; 95% CI). For the NHS algorithm, symptoms could be recorded on any of the 3 days. A Mann–Whitney *U* test was used to assess statistical significance. AUC=area under the receiver operating characteristic curve. NHS=National Health Service.

* p<0·01.

† p<0·05.

‡ Statistically different from the hierarchical Gaussian process model.

§ Statistically different from the hierarchical Gaussian process model and the logistic regression model proposed by Menni and colleagues.^7^

When used to predict signs of infection during 3 days of self-reported symptoms, the logistic regression model had a lower AUC than did the hierarchical Gaussian process model, but had a higher AUC than did the NHS algorithm (table 2). Across all timepoints, the logistic regression model had significantly lower sensitivity than did the hierarchical Gaussian process model, when considering the optimal threshold (table 2). Additionally, the logistic regression model had a non-statistically significant lower sensitivity than did the NHS algorithm model when using a window of 3 days of self-reported symptoms. For 3 days of self-reported symptoms, the specificity of the hierarchical Gaussian process model was numerically lower than the specificity of the NHS algorithm or the logistic regression model (table 2).

Only the hierarchical Gaussian process model was used in sensitivity and specificity subgroup analyses because it outperformed the other two models. In these subgroup analyses, the AUCs were similar across the first three age groups (16–39 years, 40–59 years, and 60–79 years), and were similar to that observed in the unstratified test set, but were smaller than the AUCs in individuals 80 years or older (appendix p 7). The mean sensitivities and specificities for participants aged 16–39 years had high SDs across all timepoints, suggesting that, despite its good performance, our model is less robust in identifying SARS-CoV-2-positive individuals in the younger population than in the older population (appendix p 7). For those 80 years or older, the model was accurate for detecting COVID-19, with low SDs for sensitivity and specificity for 2 days and 3 days of self-reported symptoms (appendix p 7). For 3 days of self-reported symptoms, sensitivity was 79% and specificity was 63% for those aged 60–79 years, and sensitivity was 100% and specificity was 89% for those 80 years or older (appendix p 7).

Model performance was also analysed in BMI subgroups (appendix p 8). Because of the small sample size of people with overweight or obesity in the test set (appendix p 4), we merged the two groups for the sensitivity and specificity analyses. The model's performance was similar across the BMI subgroups to that in the unstratified test set, with the exception of the underweight subgroup, in whom the model had a lower AUC for 3 days of self-reported symptoms (0·74 [95% CI 0·73–0·75]). The underweight subgroup also had a higher rate of false negatives than did the healthy weight subgroup and the newly formed overweight and obese subgroup, which translated into a sensitivity of 59% after 3 days of symptoms (appendix p 8). Stratification by sex, however, revealed no differences in model performance for the detection of COVID-19 (appendix p 8).

In a subgroup analysis based on occupation (health-care workers, who are at greater risk of infection,^16^ *vs* non-health-care workers), our model had a higher predictive power to predict COVID-19 positivity for non-health-care workers (AUC 0·81 [95% CI 0·80–0·81]), with a sensitivity of 76%, than it had for health-care workers (0·76 [0·74–0·78]), with a sensitivity of 63%, after 3 days of self-reported symptoms (appendix p 8). The higher SDs for the health-care worker metrics compared with the non-health-care worker metrics across folds highlights the low robustness of the model when used for health-care workers (appendix p 8).

We assessed feature relevance of self-reported symptoms and comorbidities in the COVID-19 prediction (appendix p 9). The most relevant symptoms to detect COVID-19 among the unstratified test set were, ordered by relevance, loss of smell, chest pain, persistent cough, shortness of breath, abdominal pain, blisters on the feet, eye soreness, and unusual muscle pain (appendix p 9). The comorbidities that showed the highest relevance to the detection of COVID-19 were heart disease, followed by kidney disease, and lung disease (appendix p 9). These comorbidities did not directly influence the detection of COVID-19, but did impact on the relevance of the symptoms for early diagnosis (appendix pp 5–6).

**Figure 1:**
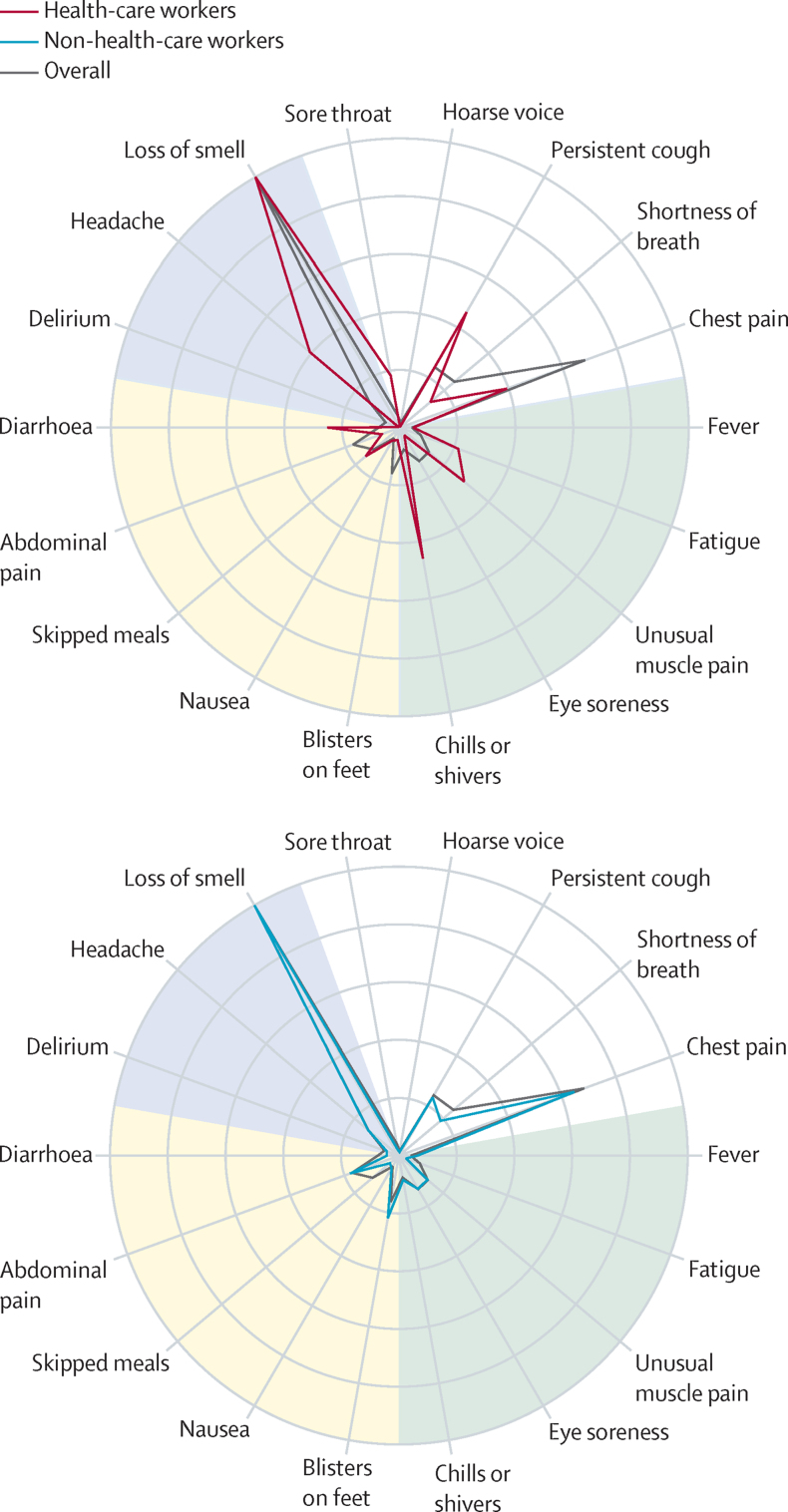
Feature relevance by occupation Symptoms are grouped according to their clinical manifestations: gastrointestinal symptoms and other symptoms (yellow sector), flu-like symptoms (green sector), neurological symptoms (purple sector), and cardiac and respiratory symptoms (white sector). The grey line represents overall symptom relevance without stratification. Points further from the centre correspond to a higher relevance. Relevance is normalised for direct interpretation.

We further analysed feature relevance according to different population subgroups (appendix p 6), also grouping symptoms into the four different groups of symptoms (appendix p 1). For both health-care workers and non-health-care workers, loss of smell was the most relevant feature for early diagnosis of COVID-19 (figure 1). However, symptoms used by the model to identify COVID-19 were significantly different in these two populations (p=0·029). Health-care workers presented with chills or shivers, persistent cough, headache, and chest pain as highly relevant symptoms, followed by unusual muscle pain, diarrhoea, fatigue, and skipped meals. Chest pain and persistent cough were symptoms (in addition to loss of smell) that were highly relevant for the detection of COVID-19 in non-health-care workers (figure 1). Blisters on the feet were relevant to the prediction of COVID-19 for non-health-care workers, despite not being a direct sign of infection (figure 1).^7^, ^11^, ^17^, ^18^, ^19^

**Figure 2:**
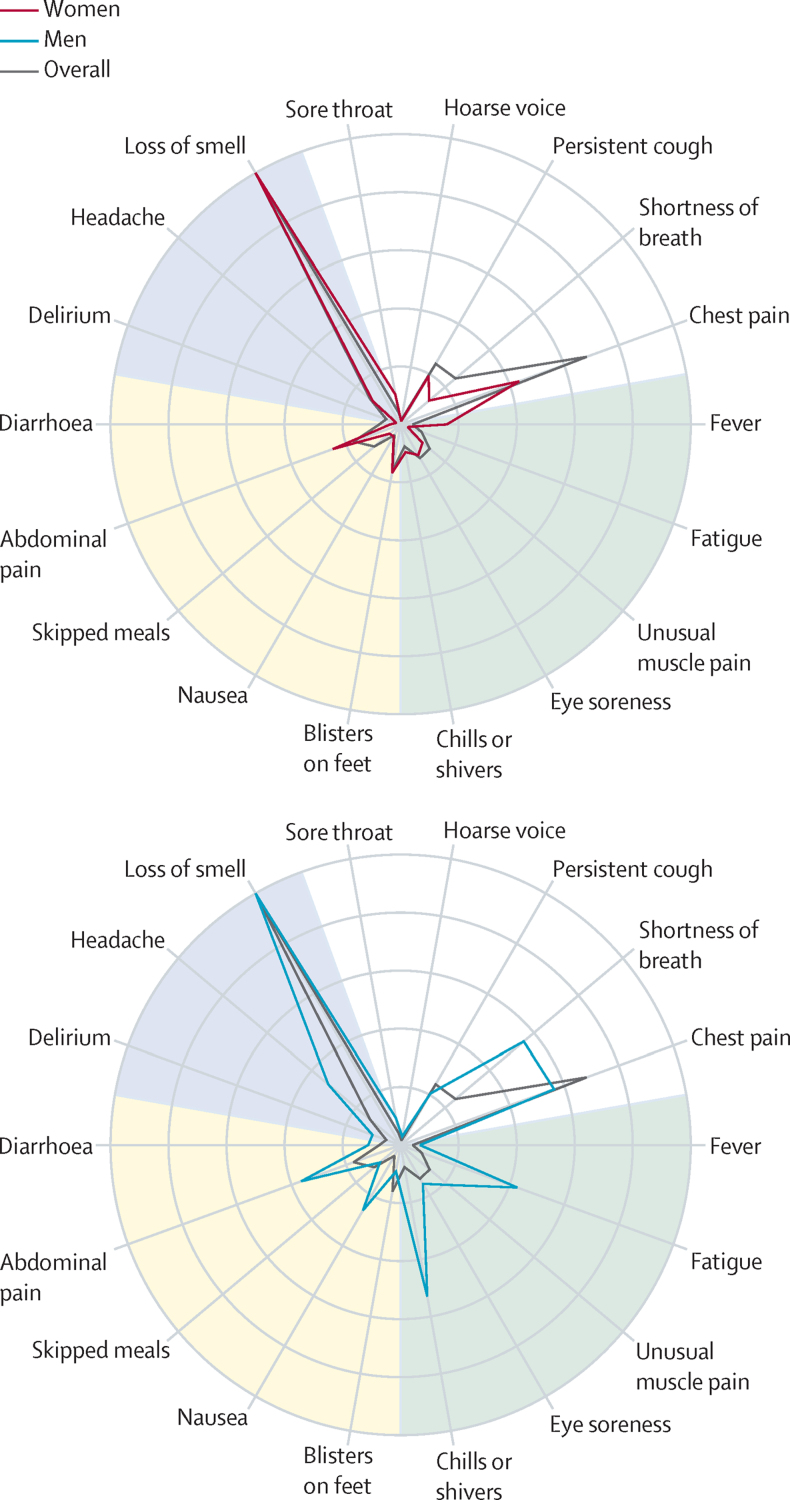
Feature relevance by sex Symptoms are grouped according to their clinical manifestations: gastrointestinal symptoms and other symptoms (yellow sector), flu-like symptoms (green sector), neurological symptoms (purple sector), and cardiac and respiratory symptoms (white sector). The grey line represents overall symptom relevance without stratification. Points further from the centre correspond to a higher relevance. Relevance is normalised for direct interpretation.

The distribution of symptom relevance was not different between the sexes (p=0·95), but shortness of breath, fatigue, and chills or shivers were more relevant features in the detection of COVID-19 for men than for women (figure 2). For both sexes, the prediction of a positive COVID-19 test was highly influenced by loss of smell, chest pain, and abdominal pain (figure 2).

**Figure 3:**
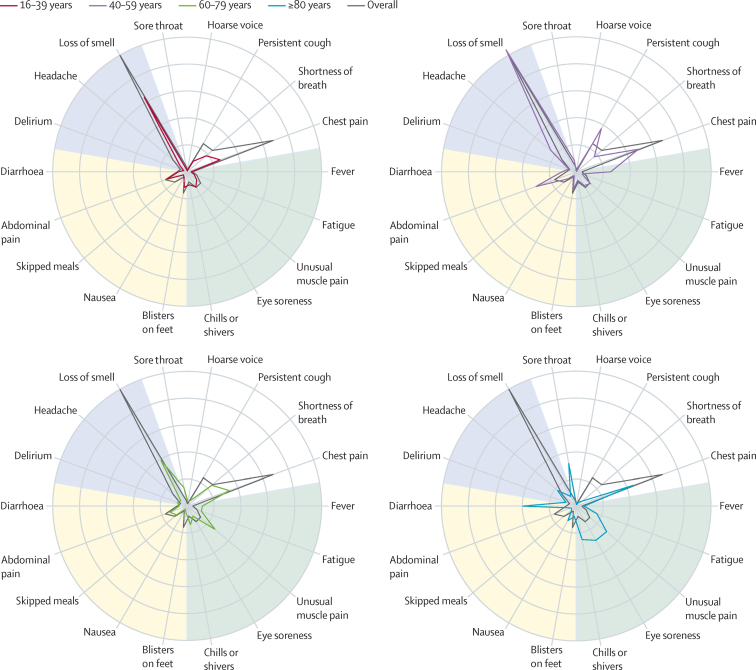
Feature relevance by age group Symptoms are grouped according to their clinical manifestations: gastrointestinal symptoms and other symptoms (yellow sector), flu-like symptoms (green sector), neurological symptoms (purple sector), and cardiac and respiratory symptoms (white sector). The grey line represents overall symptom relevance without stratification. Points further from the centre correspond to a higher relevance. Relevance is normalised for direct interpretation.

For participants aged 16–39 years, loss of smell, chest pain, abdominal pain, shortness of breath, and eye soreness were the most relevant symptoms during the initial 3 days of self-reporting (figure 3). We identified significant differences in the relevance of all symptoms between the 40–59 years age group and the 60–79 years age group (p=0·0038), and between the 40–59 years age group and the 80 years or older age group (p=0·0040; figure 3), but not between any other age groups. For individuals aged 40–59 years, persistent cough had a higher relevance to detect COVID-19 and chills or shivers had a lower relevance to detect COVID-19 compared with individuals 80 years or older. As participant age increased to 60 years or older, loss of smell began to lose relevance, and, for participants 80 years or older, loss of smell was not the most relevant feature in the detection of COVID-19 (figure 3). Specifically, for individuals aged 60–79 years, chest pain, unusual muscle pain, shortness of breath, and loss of smell were the most relevant features, whereas, for participants 80 years or older, diarrhoea, sore throat, chest pain, unusual muscle pain, eye soreness, and chills or shivers were the most relevant symptoms (figure 3).

**Figure 4:**
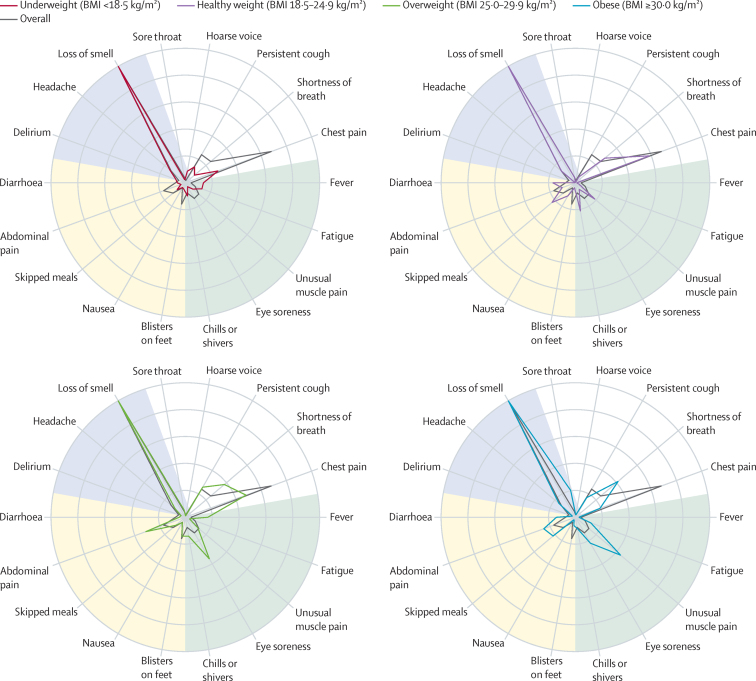
Feature relevance by BMI category Symptoms are grouped according to their clinical manifestations: gastrointestinal symptoms and other symptoms (yellow sector), flu-like symptoms (green sector), neurological symptoms (purple sector), and cardiac and respiratory symptoms (white sector). The grey line represents overall symptom relevance without stratification. Points further from the centre correspond to a higher relevance. Relevance is normalised for direct interpretation. BMI=body-mass index.

Loss of smell was consistently relevant for all BMI groups (figure 4). We did not identify any significant differences in the distribution of symptom relevance across the BMI groups (p>0·0041). Patients who were underweight did not show a specific, distinctive pattern of relevant symptoms to detect COVID-19, with only a high relevance for loss of smell and chest pain (figure 4). Conversely, for those with a healthy BMI, our model designated loss of smell, chest pain, shortness of breath, chills or shivers, skipped meals, unusual muscle pain, diarrhoea, and nausea as relevant features. Loss of smell, chest pain, shortness of breath, eye soreness, abdominal pain, persistent cough, fever, chills or shivers, and blisters on the feet were relevant for participants with overweight, whereas abdominal pain, shortness of breath, diarrhoea, unusual muscle pain, and loss of smell were relevant in the detection of COVID-19 among participants with obesity (figure 4). The uncertainty of label prediction was computed to understand how confident the model was in predicting COVID-19 positivity for age, sex, occupation, and BMI subgroups. A wider window of symptoms (3 days) led to more certain predictions for all age groups, except for patients aged 40–59 years (appendix pp 9, 11). There was no difference between the sexes in the confidence of label predictions (appendix pp 9–10). The predicted label was less confident for health-care workers than for non-health-care workers, even when using 3 days of self-reported symptoms (appendix pp 9–10). The model showed a generally high certainty of predictions across BMI subgroups, although participants in the underweight and obese subgroups (compared with the healthy weight and overweight subgroups) were predicted as COVID-19-positive with lower certainty; for participants with obesity, there was a decrease in the certainty of predictions with an increase in the number of days of self-reported symptoms (appendix pp 9, 12).

## Discussion

The early detection of COVID-19 is helpful for resource allocation during the pandemic. Here, we have proposed a Bayesian approach to identify individuals with probable COVID-19 on the basis of self-reported symptoms during 1, 2, and 3 days after symptom onset and demographic information. Using a unique, prospective dataset, we evaluated the proposed method by analysing its ability to predict COVID-19 in the early stages of infection, identify sets of symptoms that can be used to characterise early signs of infection in subgroups of the population, and consider the certainty of estimations for the model predictions to be used to direct people for testing, self-isolation, or both. This early diagnosis could then lead to better allocation of medical resources when the health-care system is severely strained by the pandemic. The proposed approach was compared with the methods currently used by the NHS and by related studies.^7^, ^9^, ^20^ Our model was effective in the identification of COVID-19 after 3 days of symptoms (AUC 0·80), with a mean sensitivity of 0·73 (SD 0·05) and a mean specificity of 0·72 (SD 0·02). Nevertheless, the model was hampered by the bias of data acquisition and requires further validation with external datasets. When compared with the other state-of-the-art diagnostic algorithms,^7^, ^9^ our proposed approach showed significantly better predictive accuracy and sensitivity. By analysing predictive AUC, sensitivity, and specificity in subgroups, we conclude that our model can be particularly relevant in the detection of early signs of COVID-19 for certain groups of the population, such as older patients. Conversely, our proposed model was less accurate in the detection of COVID-19 among health-care workers compared with non-health-care workers.

We identified loss of smell, chest pain, persistent cough, abdominal pain, blisters on the feet, eye soreness, and unusual muscle pain as the most relevant features indicating early signs of COVID-19.^7^, ^9^, ^10^, ^17^, ^18^, ^19^ From a previous study^7^ on patients symptomatic for COVID-19, skipped meals and fever were highlighted as relevant symptoms in the identification of COVID-19.^9^, ^10^, ^11^, ^20^ However, our analysis showed that these features were not relevant to early disease in the unstratified population, and so skipped meals and fever should not be considered as first-line symptoms indicating that patients should have a COVID-19 test or self-isolate. In addition, among the comorbidities reported by the participants in our study, heart disease was the most relevant to the predictions. Although patients' comorbidities did not directly affect the outcome of the model, they were included in the model as conditional variables. Therefore, the symptoms of COVID-19 reported by individuals with previous heart conditions should be further investigated and differentiated from the symptoms reported by the general population.

Using a hierarchical Gaussian process model, we further investigated the early signs of infection in subgroups of the population. Our initial results suggested that health-care workers showed distinctive features compared with non-health-care workers. For both groups, loss of smell was the most relevant feature for early diagnosis of COVID-19, but fatigue, headache, skipped meals, and unusual muscle pain were more relevant to health-care workers than to non-health-care workers.^16^ We believe that the workload faced by health-care workers during the pandemic increases both their exposure to the virus and their stress levels, which could explain the relevance of such symptoms; symptoms related to long-term stress could potentially lead to psychological symptoms that are translated as fatigue.^16^, ^21^, ^22^ Similarly, the unusual muscle pain could also be explained by long work periods in health-care settings and the physical demand of caretaking during the pandemic.^16^, ^22^ Our model also had a lower predictive power for health-care workers (AUC 0·76; 63% sensitivity) than it had for non-health-care workers (AUC 0·81; 76% sensitivity) after 3 days of self-reported symptoms. This result could be explained by the differences between these groups in feature relevance and the possibility that health-care workers experience and report symptoms in a different way to non-health-care workers. We think that current studies investigating COVID-19 symptoms could benefit from a personalised model incorporating, and trained using, participants' occupations.

By stratifying the relevance of symptoms per age group, we showed that early symptoms reported by participants from some different age groups varied.^23^ We found that loss of smell, a symptom that is being widely used to detect COVID-19, begins to lose relevance for people older than 60 years and is not a relevant feature for individuals 80 years or older. These new results suggest that the detection of early signs of COVID-19 could benefit from personalised models that factor in the age group of participants. The differences in feature relevance could also be explained by the small number of people in the age groups used, specifically older people who might be less prone to register their symptoms regularly, and fewer evident and aggressive symptoms in younger participants than in older participants.^24^, ^25^ Therefore, future research should focus on the development of sub-models targeting the specificities of the age subgroups that showed significantly different features. Nevertheless, the prediction of COVID-19 diagnosis across all the age groups had a consistently high certainty.

Despite the differences in prognosis and mortality for both sexes,^26^ we did not find any differences in the early signs of infection across sexes.

Our model's performance was similar across the BMI subgroups to that in the unstratified test set, with the exception of the underweight subgroup, in whom the model had a lower AUC for 3 days of self-reported symptoms. However, our model had highly uncertain predictions for patients with obesity, with a decrease in the likelihood of the predictions with an increase in the number of timepoints. This result could partly be explained by other underlying medical conditions of participants with overweight that could hamper the correct assessment of early signs of infection. The number of participants with obesity in our study population was lower than the number of participants in any other BMI category, which compromised the ability of our model to correctly describe the early signs of infection in this subpopulation.

Our study had several strengths. First, it is unique; to our knowledge, this study was the first to attempt to detect early signs of COVID-19 using self-reported symptoms. Second, the models presented in this study were trained on a large population of 182 991 participants and subsequently validated on a fully independent sample of 15 049 individuals. Therefore, the sample size of our data supports the generalisability and robustness of our approach. The heterogeneity in the demographics of included individuals and the broad spectrum of symptoms reported also resulted in a generalisable model. Third, the prospective nature of symptoms logging in this study will potentially allow us to change the model design and further improve the proposed approach. We could then develop personalised models according to various population strata, such as age groups and occupation, and validate them in future analyses. Fourth, our proposed approach has a temporal component, which did not require the concatenation of symptoms across timepoints. This aspect ensured that the sequential presentation of symptoms was not neglected, while predicted labels of participants with a different number of timepoints were still generated. Finally, the information regarding the uncertainty of the predicted labels for each subpopulation can also be used as a surrogate measure of the likelihood of an individual to be positive for SARS-CoV-2 across the different timepoints, a major advantage when used in real-life scenarios.

Our study also had limitations. First, the self-reporting nature of the data, particularly the symptoms, could have negatively affected the performance of the models. Given that the models rely on prospective data collection to work, it was necessary that the participants recalled the exact symptom trajectory of their first 3 days and the symptoms onset, which might not have always been possible. The symptoms reported might also have been overestimated, both in intensity and time, by the participants. Furthermore, the absence of clinical scales for symptoms reporting and assessment can impact the understanding and translation of the symptoms profile into the clinical environment. These factors can compromise the models' performance, limiting their use as clinical tools. To overcome these limitations, complementary measurements obtained by wearable sensors and devices could be included as features. In fact, such devices have proven successful as clinical proxies of participants' conditions and are viable solutions in assessing and validating self-reported symptoms.^27^, ^28^

Second, because of the method used for data acquisition—the mobile phone app—the study population was also skewed towards a younger population. Therefore, the translation of the proposed approach to other populations will require a detailed analysis of the participants' demographics. Nevertheless, thanks to the flexibility and the non-parametric nature of our model, we believe that model performance will not be negatively impacted, even if the relevant features change.

Third, the assessment of symptoms relevance could have potentially been impacted by the sample size of the different population strata. To reduce the effect of small samples sizes, we reduced the granularity of the BMI subgroups. We addressed these limitations by doing an extensive validation on an independent sample, which included a bootstrapping scheme to reduce sample bias and compensate for different symptom prevalence across individuals and population strata.

Fourth, all the analyses presented in this study were done on the UK population, hence limiting the generalisability of our conclusions, as features of the study population can differ between countries. In fact, some of the population features considered for model estimation, namely obesity rates, age, comorbidities, and infection risk for health-care workers during the pandemic, could vary strongly between countries, including several low-income and middle-income countries.^29^, ^30^ Also, we did not do any specific analysis considering the ethnicity of the participants as a possible covariate in the model or confounding effect. Future work should focus on the validation of the proposed approach on different populations with different demographic features.

Finally, the guidelines for testing according to the available resources can be considered another key limitation of this study. Given that the likelihood of being offered a test in the UK is strongly dependent on the symptoms used for reference by the NHS,^11^ an individual's occupation being considered among other factors, the outcome of the test itself can be biased. Similarly, different actions for the mitigation of COVID-19 across countries could also impact the manifestation of the disease and the test used as a reference to define SARS-CoV-2 positivity.

Early detection of SARS-CoV-2-infected individuals is crucial to contain the spread of the COVID-19 pandemic and efficiently allocate medical resources. In this study, we proposed a tailored hierarchical Gaussian process model to predict the early signs of infection using self-reported symptoms. This model allows us to refer individuals for testing and self-isolation even when only early symptoms are observed. In the future, our proposed model can integrate additional features, such as clinically relevant measures, to improve and reduce the bias associated with self-reported inputs.

## Data sharing

Data collected in the COVID-19 Symptom Study smartphone app are being shared with other health researchers through the NHS-funded Health Data Research UK and Secure Anonymised Information Linkage consortium, housed in the UK Secure Research Platform (Swansea, UK). Anonymised data are available to be shared with researchers (with no date restrictions), according to their protocols, in the public interest (https://web.www.healthdatagateway.org/dataset/594cfe55-96e3-45ff-874c-2c0006eeb881). The code for the hierarchical Gaussian process model is freely available at https://gitlab.com/KCL-BMEIS/covid-zoe/prediction-gp.

## Declaration of interests

ATC reports personal fees from Pfizer, Bayer Pharma, and Boehringer Ingelheim, outside the submitted work. CJS reports grants from the Chronic Disease Research Foundation, during the conduct of the study. JCP, LP, JW, and TS report other (work) and consultancy from ZOE, during the conduct of the study. CHS reports grants from the Alzheimer's Society, during the conduct of the study. DAD reports grants from the National Institutes of Health during the conduct of the study and has previously served as a co-investigator on an unrelated trial supported by ZOE. RD reports grants from the Department of Health and Social Care, during the conduct of the study, and personal fees from ZOE, outside the submitted work. SO reports grants from the Wellcome Trust, Innovate UK Research and Innovation, and the Chronic Disease Research Foundation, during the conduct of the study. All other authors declare no competing interests.
