# Supplementary material for: Early detection of COVID-19 in the UK using self-reported symptoms: a large-scale, prospective, epidemiological surveillance study
Source: Lancet Digit Health. 2021 Jul 29;3(9):e587–98. doi: 10.1016/S2589-7500(21)00131-X (PMC8321433; doi:10.1016/S2589-7500(21)00131-X)
Supplement: Supplementary appendix [file mmc1.pdf]

THE LANCET
Digital Health

Supplementary appendix

This appendix formed part of the original submission and has been peer reviewed. We post it as supplied by the authors.

Supplement to: Canas LS, Sudre CH, Capdevila Pujol J, et al. Early detection of COVID-19 in the UK using self-reported symptoms: a large-scale, prospective, epidemiological surveillance study. *Lancet Digit Health* 2021; published online July 29. https://doi.org/10.1016/S2589-7500(21)00131-X.

Supplementary material

Symptoms description and demographic information

The symptoms included in this study differ from the symptoms currently acquired using the app, which have been adapted to account for the report of additional symptoms. All the symptoms are binary encoded (0 – not experienced, and 1 – experienced), with exception of fatigue and shortness of breath that are encoded using a severity scale. The information about existing comorbidities, as well as demographic information such as gender, age and occupation, here considering only healthcare workers, are available for all subjects. For the purposes of this research, the study population was limited to participants from the UK, who consent to participate in the study and who provide sufficient data. The consent is given when signing up in the app, where the users are informed that the data will be shared for research purposes. All the subjects have been labelled according to the self-reported PCR test result as positive or negative for SARS-CoV-2, which is then used as diagnostic criteria for both model training and evaluation. In addition to the self-reported symptoms and the personal characteristics included in the model, we also considered as a feature the prevalence ratio of COVID-19 associated with the subjects' geographical area. This information was retrieved from using the approach proposed by Varsavsky et al., that uses symptom-based and swab-based information to predict the prevalence per Upper Tier Local Authority (UTLA).(9) Note that despite the prevalence ratio being included in the model, the geographical area was previously anonymised.

Supplementary Table 1. List of symptom questions asked by the COVID Symptom Study app. Questions were: Do you have (symptom)? Answers were yes/no, unless indicated otherwise.

Symptom	***COVID Symptom Study app question***
Fever	Fever (at least 37.8C or 100F). 0 – No; 1 – Yes;
Persistent Cough	Persistent cough (coughing a lot for more than an hour or 3 or more coughing episodes in 24 hours). 0 – No; 1 - Mild; 2 – Medium; 3 – Severe.
Fatigue	Unusual fatigue… (no; mild fatigue; severe fatigue/ I struggle to get out of bed). 0 – No Fatigue; 1 - Fatigue
Shortness of breath	Shortness of breath or trouble breathing. 0 – No; 1 - yes mild symptoms/ slight shortness of breath during ordinary activity; 2 - yes significant symptoms/ breathing is comfortable only at rest; 3 - yes, severe symptoms/ breathing is difficult even at rest).
Loss of smell	Loss of smell / taste. 0 – No loss of smell; 1 – loss of smell.
Hoarse Voice	Unusually hoarse voice. 0 – No; 1 - Yes
Chest Pain	Unusual chest pain or tightness in your chest. 0 – No; 1 - Yes
Abdominal Pain	Unusual abdominal pain or stomach ache. 0 – No; 1 - Yes
Diarrhoea	Diarrhoea. 0 – No; 1 - Yes
Delirium	Confusion, disorientation or drowsiness. 0 – No; 1 - Yes
Eye Soreness	Do your eyes have any unusual eye-soreness or discomfort (e.g. light sensitivity, excessive tears, or pink/red eye)? 0 – No; 1 - Yes
Skipped meals	Skipping meals. 0 – No; 1 - Yes
Headache	Headache. 0 – No; 1 - Yes
Nausea	Nausea or vomiting. 0 – No; 1 - Yes
Dizziness	Dizziness or light-headedness. 0 – No; 1 - Yes
Sore Throat	Sore or painful throat. 0 – No; 1 - Yes
Unusual muscle pain	Unusual strong muscle pains or aches. 0 – No; 1 - Yes
Red Welts in Face and Lips	Raised, red, itchy welts on the skin or sudden swelling of the face or lips. 0 – No; 1 - Yes
Blisters on feet	Red/purple sores or blisters on your feet, including your toes. 0 – No; 1 - Yes

Supplementary Table 2. Symptoms grouping. The symptoms are group according to their clinical manifestations. Flu group refers to the symptoms mimicking flu symptoms.

Group	***Symptoms***
Gastrointestinal and others	diarrhoea, abdominal pain, skipped meals, nausea and others (blisters on the feet)
Flu like	fever, fatigue, chills or shivers, eyes soreness, unusual muscle pain
Neurological	delirium, headache and loss of smell
Cardiac and Respiratory	shortness of breath, persistent cough and chest pain, hoarse voice, sore throat

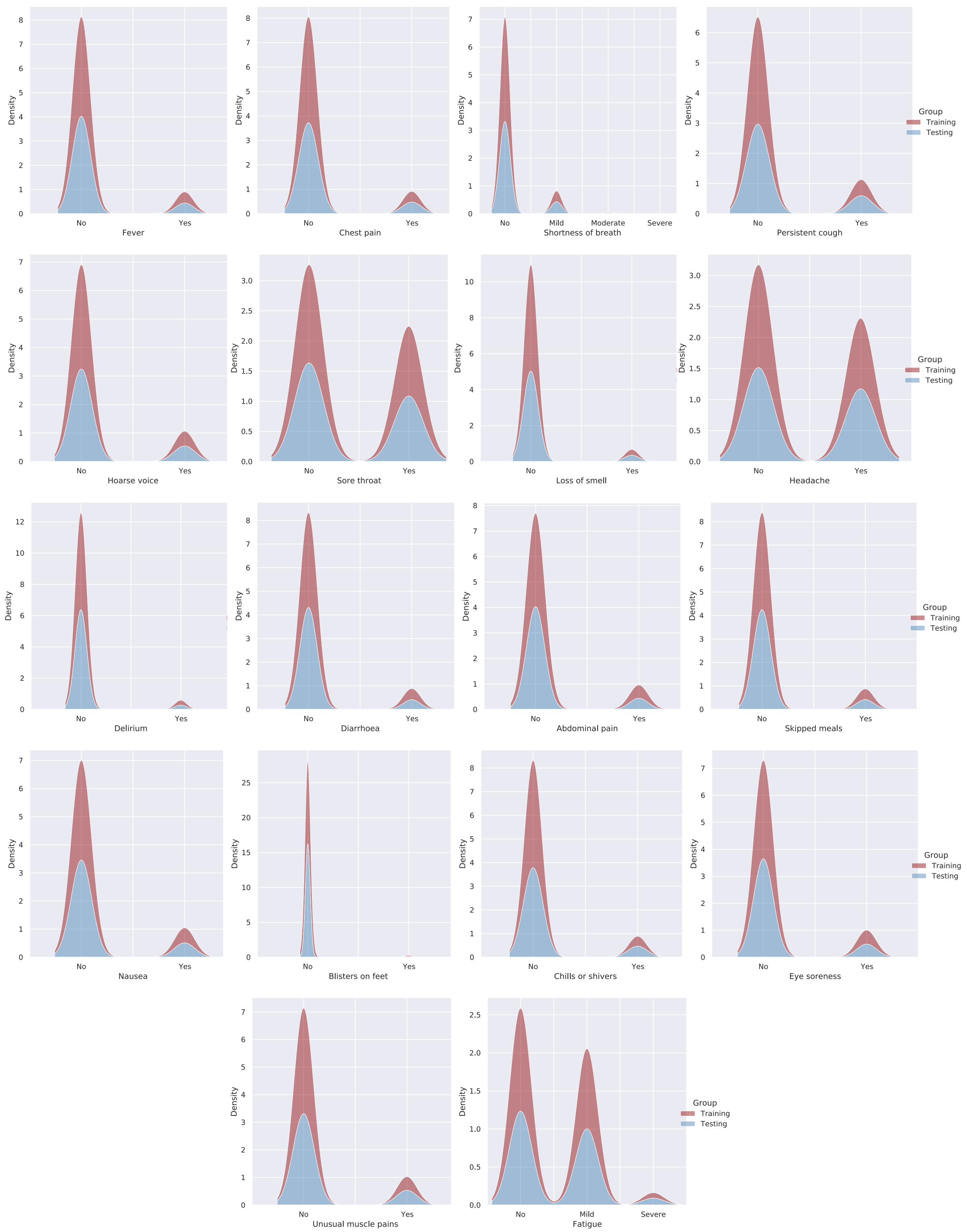

Supplementary Figure 1. Distribution of symptoms for the two datasets in analysis: training, validation and testing. The distribution of the symptoms is comparable across datasets.

Supplementary Table 3. Demographic information per age group in the study population. The population is separated by positive and negative outcomes of the SARS-CoV-2 testing. Information is presented for the training dataset composed by data from 29th of April 2020 to the 14th of October 2020, and an independent dataset, testing data, which includes the samples reported from the 15th of October 2020 to the 30th of November 2020. Yo denotes years old.

			**SARS-CoV-2 Positive**			**SARS-CoV-2 Negative**		
			**1 day**	**2 days**	**3 days**	**1 day**	**2 days**	**3 days**
**16-39 yo**	**Number (%)**	*Training set*	607 (1.6)	330 (2.1)	341 (2.6)	37706 (98.4)	15236 (97.9)	12844 (97.4)
		Testing set	248 (7.7)	169 (12.3)	152 (14.4)	2959 (92.3)	1207 (87.7)	903 (85.6)
	**Age (years)**	*Training set*	29.2 (6.3)	29.4 (6.2)	29.8 (6.2)	32.4 (5.0)	32.7 (4.9)	32.7 (4.9)
		Testing set	31.8 (5.7)	32.2 (5.5)	32.1 (5.6)	32.7 (4.9)	32.8 (4.8)	33.0 (4.7)
**40-59 yo**	**Number (%)**	*Training set*	993 (1.4)	542 (1.7)	526 (1.9)	71071 (98.6)	30430 (98.3)	26864 (98.1)
		Testing set	632 (8.2)	406 (11.6)	372 (14.1)	7086 (91.8)	3086 (88.4)	2272 (85.9)
	**Age (years)**	*Training set*	50.3 (5.6)	50.3 (5.7)	50.4 (5.6)	49.7 (5.7)	49.7 (5.7)	49.7 (5.7)
		Testing set	50.7 (5.5)	50.6 (5.5)	50.7 (5.7)	50.1 (5.6)	50.1 (5.6)	50.1 (5.6)
**60-79 yo**	**Number (%)**	*Training set*	359 (1.0)	219 (1. 5)	211 (1.6)	35216 (99.0)	14620 (98.5)	12898 (98.4)
		Testing set	275 (6.8)	175 (9.4)	153 (10.9)	3799 (93.2)	1682 (90.6)	1256 (89.1)
	**Age (years)**	*Training set*	65.8 (4.7)	65.6 (4.6)	65.2 (4.5)	66.1 (4.6)	66.0 (4.6)	66.0 (4.5)
		Testing set	65.6 (4.5)	65.4 (4.2)	65.9 (4.1)	66.0 (4.6)	65.9 (4.4)	65.9 (4.4)
**≥80 yo**	**Number (%)**	*Training set*	6 (1.3)	2 (1.2)	4 (2.8)	497 (98.8)	169 (98.8)	140 (97.2)
		Testing set	3 (6.6)	2 (10.0)	2 (20.0)	47 (94.0)	18 (90.0)	8 (80.0)
	**Age (years)**	*Training set*	83.7 (3.1)	82.5 (0.5)	83.5 (3.8)	82.2 (2.6)	82.1 (2.7)	81.9 (2.4)
		Testing set	83.3 (3.4)	85.0 (3.0)	85.0 (3.0)	81.9 (2.4)	82.8 (3.3)	81.6 (1.5)

Supplementary Table 4. **Demographic information per BMI group in the study population.** The population is separated by positive and negative outcomes of the SARS-CoV-2 testing. Information is presented for the training dataset composed by data from 29th of April 2020 to the 14th of October 2020, and an independent dataset, testing data, which includes the samples reported from the 15th of October 2020 to the 30th of November 2020. BMI denotes body mass index.

			**SARS-CoV-2 Positive**			**SARS-CoV-2 Negative**		
			**1 day**	**2 days**	**3 days**	**1 day**	**2 days**	**3 days**
**Underweight**	**Number (%)**	*Training set*	114 (1.3)	62 (1.6)	52 (1.6)	8988 (98.7)	3873 (98.4)	3267 (98.4)
		Testing set	60 (6.8)	38 (9.5)	34 (11.4)	825 (93.2)	362 (90.5)	264 (88.6)
	**BMI (kg/m^2)**	*Training set*	16.6 (1.4)	16.7 (1.3)	16.8 (1.2)	16.5 (1.3)	16.5 (1.3)	16.5 (1.3)
		Testing set	16.8 (1.1)	16.8 (1.0)	16.9 (1.1)	16.6 (1.2)	16.6 (1.2)	16.6 (1.3)
**Healthy**	**Number (%)**	*Training set*	702 (1.3)	381 (1.7)	387 (2.0)	52455 (98.7)	22153 (98.3)	19391 (98.0)
		Testing set	383 (6.9)	246 (9.8)	225 (12.0)	5160 (93.1)	2255 (90.2)	1653 (88.0)
	**BMI (kg/m2)**	*Training set*	21.6 (1.8)	21.6 (1.7)	21.5 (1.7)	21.5 (1.8)	21.5 (1.8)	21.5 (1.8)
		Testing set	21.4 (1.8)	21.4 (1.8)	21.5 (1.9)	21.5 (1.8)	21.5 (1.8)	21.5 (1.8)
**Overweight**	**Number (%)**	*Training set*	535 (1.3)	308 (1.8)	317 (2.1)	40242 (98.7)	16807 (98.2)	14594 (97.9)
		Testing set	332 (7.9)	231 (11.9)	208 (14.1)	3883 (92.1)	1711 (88.1)	1267 (85.9)
	**BMI (kg/m2)**	*Training set*	26.6 (1.4)	26.6 (1.4)	26.7 (1.4)	26.8 (1.5)	26.8 (1.5)	26.7 (1.5)
		Testing set	27.0 (1.5)	27.0 (1.5)	26.8 (1.4)	26.7 (1.4)	26.7 (1.4)	26.7 (1.5)
**Obese**	**Number (%)**	*Training set*	610 (1.4)	338 (1.9)	324 (2.1)	42366 (98.6)	17441 (98.1)	15318 (97.9)
		Testing set	379 (8.7)	234 (12.5)	207 (14.3)	3983 (91.3)	1643 (87.5)	1240 (85.7)
	**BMI (kg/m2)**	*Training set*	35.1 (5.2)	35.1 (5.2)	35.0 (5.3)	35.4 (5.4)	35.2 (5.4)	35.3 (5.3)
		Testing set	35.2 (5.5)	35.8 (5.8)	35.6 (5.7)	35.3 (5.4)	35.2 (5.3)	35.4 (5.6)

Models definition

NHS algorithm:

The NHS considers three symptoms as the main indicators of COVID-19 positive infection: loss of smell, fever and persistent cough (28). To infer the subject status whilst considering the current NHS algorithm, we selected these three symptoms from the 19 self-reported symptoms of the COVID-19 Study app. The sum of the maximum of the symptoms for each period of time is used to predict the outcome of the COVID-19 test. A logistic function is then used to transform the obtained results into a probabilistic prediction.

Logistic Regression:

The logistic regression was proposed by Menni *et al.,* (29) to identify positive COVID-19 patients based on a subset of symptoms and their demographic information. The proposed model successfully identified 76% of the cases of COVID-19 infection. However, the logistic regression (LR) was trained using the peak of the symptoms, which might be outside of the window used to early detect signs of infection. Here, we test the validity of this model when using the self-reported symptoms outside of their intensity maximum.
Therefore, we define a LR function, Equation 1, considering the age, gender, loss of smell, persistent cough, fatigue and skipped meals as covariates of the model. The weights of the model, $\beta_f\ with\ f \in [0,6]$, are optimised using the sum of the maximum of the reported symptoms.

$$y = -\beta_0 - (\beta_1 \times \text{Age}) + (\beta_2 \times \text{Gender}) + (\beta_3 \times \text{Loss of smell}) + (\beta_4 \times \text{Persistent cough}) + (\beta_5 \times \text{Fatigue}) + (\beta_6\ \text{Skipped meals})$$

Equation 1

As a result, three independent models are optimised for the three timestamps considered: 1, 2 and 3 days of self-reported symptoms. The model is defined and optimised using the open-source Python toolbox Scikit-learn (30).

Hierarchical Gaussian Process:

We propose a Hierarchical Gaussian process model (HGP) (30), with a tailored kernel function to evaluate the likelihood of a subject being infected based on the initial three days of symptoms. GP is commonly used to perform long term predictions and has been shown to improve performances as the number of samples increases.
The model, in Equation 2, is defined as the contributions of the underlying time-series of the data. Firstly, f_n describes the time-series of the population composed of *n* samples (subjects). This function encodes the demographic information, namely the age, gender and occupation. Thus, $k_{f_n}(t,t') = k_{Age}(t,t') + k_{Gender}(t,t') + k_{Occupation}(t,t')$. The aforementioned covariance functions have a squared exponential structure. To combine different inputs from a same subject s_n, a second Gaussian process is used with mean $f_n(t)$. The covariance function in this second GP encodes the subject specific information, such as BMI, comorbidities and prevalence ratio from her/his geographical area. Therefore, $k_s(t,t') = k_{BMI}(t,t') \times k_{Comorbidities}(t,t') \times k_{prevalence}(t,t')$. Both the covariance function encoding the prevalence and BMI have a squared exponential structure, whereas the comorbidities are encoded using an arc cosine of order 1. Lastly, the different time-points and their correlation across subjects are explained by third GP with mean $s_n(t)$. Given that this last function aims to correlate the self-reported symptoms with the expected progression of the disease during the first three days, the covariance function $k_{s'}(t,t')$ is used to encode the different symptoms as $k_{s'}(t,t') = k_{Age\_g}(t,t') \times k_{Symptoms}(t,t')$. Similarly to the previous GP, the covariance function encoding the age group is defined by a squared exponential kernel, whilst the symptoms are encoded by an arc cosine function of order 2. The arc cosine function is chosen to encode the more complex and heterogenous structure of data of the symptoms due to its similar behaviour to a multi-layer perceptron.

The main advantage of this model lies in its elegance given by its own linearity. Therefore, despite the complex structure of three GP model, two points on the function $f_n(t)$ are jointly Gaussian distributed with zero mean and covariance $k_{f_n}(t,t') + k_s(t,t') + k_{s'}(t,t')$. Furthermore, two points from different subjects and time-points are jointly distributed with covariance $k_{s'}(t,t')$. Thus, given a set of *n* samples with *i* subjects and *j* time-points, the likelihood can be defined as in Equation 3, with covariance function defined as in Equation 4.

$$p(y_n|X_n,\theta) := \begin{cases} f_n(t) = \left(0, k_{f_n}(t,t')\right) \\ s_n(t) = \left(f_n(t), k_s(t,t')\right) \\ s'_n(t) = \left(s_n(t), k_{s'}(t,t')\right) \end{cases} \qquad \text{Equation 2}$$

$$p(y_n|X_n,\theta) = \mathcal{GP}(0,\Sigma_\mathrm{n}), \qquad \text{Equation 3}$$

$$\Sigma_\mathrm{n} = \begin{cases} K'_s\left(t_{n,i,j}, t'_{n,i,j}\right) + K_s\left(t_{n,i,j}, t_{n',i',j'}\right) + K_f\left(t_{n,i,j}, t_{n',i',j'}\right) + \sigma I \quad \text{if } j = j' \\ K_s\left(t_{n,i,j}, t_{n',i',j'}\right) + K_f\left(t_{n,i,j}, t_{n',i',j'}\right) \quad \text{if } i = j' \\ K_f\left(t_{n,i,j}, t_{n',i',j'}\right) \text{ otherwise} \end{cases}$$

Equation 4

Note that the model encodes both the correlation between symptoms across subjects and also across time-points. As a result, a single model can be used to predict the COVID-19 status for an unseen subject using a flexible number of time-points. The probabilistic outcome of the predicted label can then be used a proxy of a clinical diagnosis, signalising the subject for either a PCR test or self-isolation while waiting for SARS-CoV-2 test results.

The relevance of the features is obtained from the optimised weights of the arc cosine kernel function, which encodes the importance of the several symptoms in the estimation of the kernel matrix.

Stratification of the features relevance

Four groups are consider as detailed below:

a) Healthcare occupation - it encodes the risk of the subjects to be exposed to COVID-19 according to their job or daily occupation. This covariate is then defined positive for healthcare workers (HCW) and null for non-healthcare workers (NHCW). All the subjects are labelled as HCW if they have an occupation connected to the healthcare system and interact with or treat people exposed to the virus. Similarly, all the people that have contact with a healthcare worker are also labelled HCW, since they are likewise exposed to risk factors that can lead to COVID-19 infection. Conversely, subjects that have not to interact with or to treat patients are defined as NHCW, since they are not directly at the risk to be exposed to the virus, hence less prone to be infected;
b) Gender – it encodes the two main sexes – female and male;
c) Age group – it stratifies the population according to their age. Four groups are defined as: 16 – 39 years old, 40 – 59 years old, 60 - 79 years old and above 80 years old.
d) BMI group – it stratifies the population according to their BMI. Four groups are included in this analysis, namely underweight (<18.5kg/m2), healthy (18.5-24.9kg/m2), overweight (25.0-29.9kg/m2), and obese (≥30kg/m2).

Prediction of COVID-19 positive testing

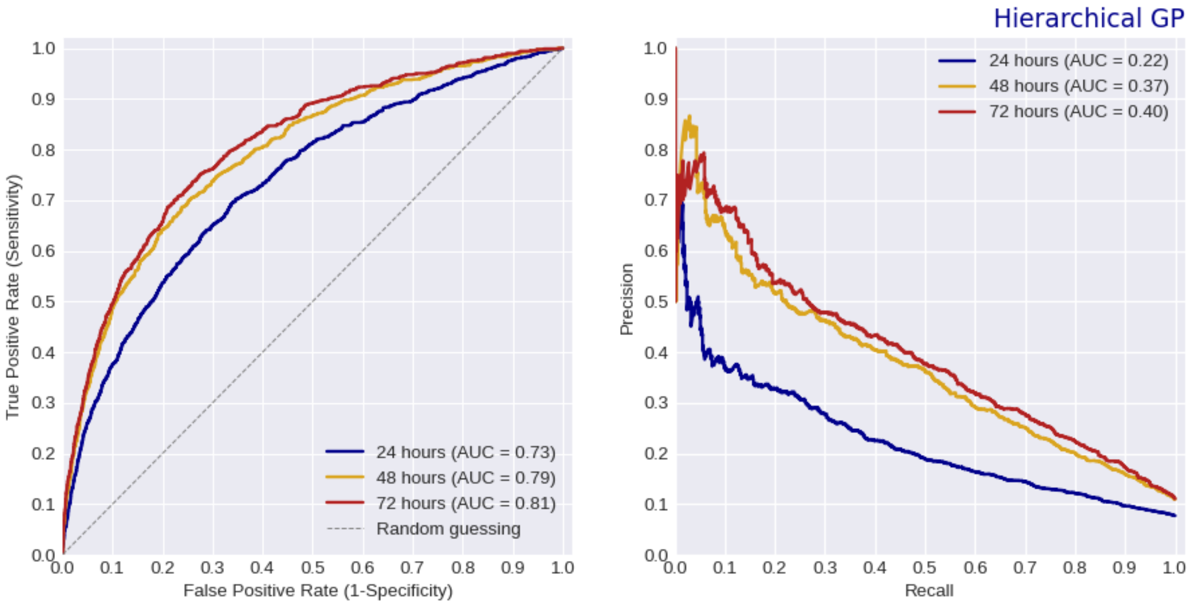

Supplementary Figure 2. Evaluation of HGP model for early COVID-19 detection. Left panel: ROC-AUC are presented for one day (24 hours), two days (48 hours) and three days (72 hours) of self-reported symptoms, used to predict the outcome of COVID-19 test. The shadow area encodes the standard deviation across folds. Right panel: Precision and Recall curves are presented for the same timestamps.

Supplementary Table 5. Performance of the model per age group. Mean and standard deviation (values in parenthesis) are computed across the 5-fold. 95% Confidence interval (CI).

		Sensitivity	Specificity	ROC-AUC
		**Optimal Threshold**	**Optimal Threshold**	
**1 Day**	**16 – 39**	0.66 (0.10) [95% CI 0.57-0.74]	0.67 (0.12) [95% CI 0.57-0.78]	0.72 (<0.01) [95% CI 0.72-0.72]
	**40 - 59**	0.70 (0.04) [95% CI 0.67-0.74]	0.63 (0.06) [95% CI 0.58-0.69]	0.72 (0.01) [95% CI 0.71-0.73]
	**60 - 79**	0.76 (0.03) [95% CI 0.74-0.79]	0.56 (0.06) [95% CI 0.50-0.61]	0.73 (0.01) [95% CI 0.72-0.74]
	**≥ 80**	0.83 (0.24) [95% CI 0.59-1.00]	0.66 (0.17) [95% CI 0.49-0.83]	0.82 (0.08) [95% CI 0.74-0.90]
**2 Days**	**16 – 39**	0.67 (0.11) [95% CI 0.58-0.77]	0.73 (0.15) [95% CI 0.60-0.86]	0.78 (0.01) [95% CI 0.77-0.78]
	**40 - 59**	0.75 (0.05) [95% CI 0.70-0.79]	0.68 (0.06) [95% CI 0.63-0.74]	0.78 (0.01) [95% CI 0.77-0.79]
	**60 - 79**	0.73 (0.09) [95% CI 0.65-0.81]	0.63 (0.12) [95% CI 0.52-0.73]	0.77 (0.01) [95% CI 0.76-0.78]
	**≥ 80**	1.00 (0.05) [95% CI 0.95-1.00]	0.90 (0.06) [95% CI 0.84-0.96]	0.93 (0.04) [95% CI 0.89-0.97]
**3 Days**	**16 – 39**	0.70 (0.10) [95% CI 0.60-0.79]	0.71 (0.16) [95% CI 0.57-0.85]	0.78 (0.01) [95% CI 0.78-0.79]
	**40 - 59**	0.73 (0.03) [95% CI 0.71-0.76]	0.67 (0.08) [95% CI 0.60-0.74]	0.80 (0.01) [95% CI 0.79-0.81]
	**60 - 79**	0.79 (0.03) [95% CI 0.76-0.82]	0.63 (0.08) [95% CI 0.55-0.70]	0.78 (0.01) [95% CI 0.78-0.79]
	**≥ 80**	1.00 (0.05) [95% CI 0.95-1.00]	0.89 (0.07) [95% CI 0.82-0.94]	0.93 (0.05) [95% CI 0.88-0.98]

Supplementary Table 6. Performance of the model per BMI group. Mean and standard deviation (values in parenthesis) are computed across the 5-folds. 95% Confidence interval (CI).

		Sensitivity	Specificity	ROC-AUC
		Optimal Threshold	Optimal Threshold	
1 Day	Underweight	0.78 (0.06) [95% CI 0.73-0.83]	0.50 (0.09) [95% CI 0.42-0.58]	0.72 (0.01) [95% CI 0.71-0.73]
	Healthy	0.66 (0.12) [95% CI 0.56-0.77]	0.68 (0.12) [95% CI 0.54-0.81]	0.75 (<0.01) [95% CI 0.75-0.75]
	Overweight/Obese	0.77 (0.06) [95% CI 0.72-0.83]	0.56 (0.09) [95% CI 0.48-0.64]	0.74 (<0.01) [95% CI 0.74-0.75]
2 Days	Underweight	0.71 (0.15) [95% CI 0.58-0.84]	0.65 (0.15) [95% CI 0.51-0.78]	0.75 (0.01) [95% CI 0.74-0.76]
	Healthy	0.72 (0.07) [95% CI 0.66-0.78]	0.70 (0.07) [95% CI 0.64-0.77]	0.79 (<0.01) [95% CI 0.79-0.80]
	Overweight/Obese	0.71 (0.08) [95% CI 0.64-0.78]	0.69 (0.06) [95% CI 0.64-0.75]	0.78 (<0.01) [95% CI 0.77-0.79]
3 Days	Underweight	0.59 (0.16) [95% CI 0.45-0.73]	0.76 (0.17) [95% CI 0.61-0.90]	0.74 (0.01) [95% CI 0.73-0.75]
	Healthy	0.72 (0.06) [95% CI 0.66-0.77]	0.73 (0.07) [95% CI 0.67-0.79]	0.80 (<0.01) [95% CI 0.80-0.81]
	Overweight/Obese	0.70 (0.10) [95% CI 0.61-0.79]	0.71 (0.10) [95% CI 0.62-0.79]	0.78 (<0.01) [95% CI 0.78-0.79]

Supplementary Table 7. Performance of the model per gender. Mean and standard deviation (values in parenthesis) are computed across the 5-folds. 95% Confidence interval (CI).

		Sensitivity	Specificity	ROC-AUC
		Optimal Threshold	Optimal Threshold	
1 Day	Female	0.77 (0.08) [95% CI 0.70-0.84]	0.56 (0.09) [95% CI 0.48-0.64]	0.73 (<0.01) [95% CI 0.73-0.73]
	Male	0.74 (0.04) [95% CI 0.70-0.78]	0.62 (0.05) [95% CI 0.57-0.66]	0.74 (0.01) [95% CI 0.74-0.75]
2 Days	Female	0.75 (0.08) [95% CI 0.69-0.82]	0.65 (0.12) [95% CI 0.54-0.75]	0.78 (<0.01) [95% CI 0.78-0.79]
	Male	0.76 (0.06) [95% CI 0.70-0.81]	0.65 (0.08) [95% CI 0.59-0.72]	0.79 (0.01) [95% CI 0.78-0.80]
3 Days	Female	0.72 (0.06) [95% CI 0.67-0.76]	0.74 (0.06) [95% CI 0.68-0.79]	0.80 (<0.01) [95% CI 0.80-0.81]
	Male	0.77 (0.05) [95% CI 0.73-0.82]	0.66 (0.08) [95% CI 0.59-0.73]	0.80 (0.01) [95% CI 0.79-0.81]

Supplementary Table 8. Performance of the model per occupation. Mean and standard deviation (values in parenthesis) are computed across the 5-folds. HCW: Healthcare workers, NHCW: non-healthcare workers. 95% Confidence interval (CI).

		Sensitivity	Specificity	ROC-AUC
		Optimal Threshold	Optimal Threshold	
1 Day	NHCW	0.79 (0.05) [95% CI 0.74-0.83]	0.54 (0.09) [95% CI 0.46-0.63]	0.74 (<0.01) [95% CI 0.74-0.74]
	HCW	0.75 (0.15) [95% CI 0.61-0.88]	0.46 (0.11) [95% CI 0.36-0.56]	0.73 (0.01) [95% CI 0.72-0.74]
2 Days	NHCW	0.72 (0.05) [95% CI 0.68-0.76]	0.72 (0.05) [95% CI 0.67-0.76]	0.79 (<0.01) [95% CI 0.79-0.79]
	HCW	0.63 (0.13) [95% CI 0.52-0.74]	0.75 (0.12) [95% CI 0.64-0.86]	0.76 (0.01) [95% CI 0.75-0.77]
3 Days	NHCW	0.76 (0.04) [95% CI 0.73-0.79]	0.70 (0.05) [95% CI 0.66-0.74]	0.81 (<0.01) [95% CI 0.80-0.81]
	HCW	0.63 (0.11) [95% CI 0.54-0.73]	0.74 (0.13) [95% CI 0.63-0.85]	0.76 (0.02) [95% CI 0.75-0.77]

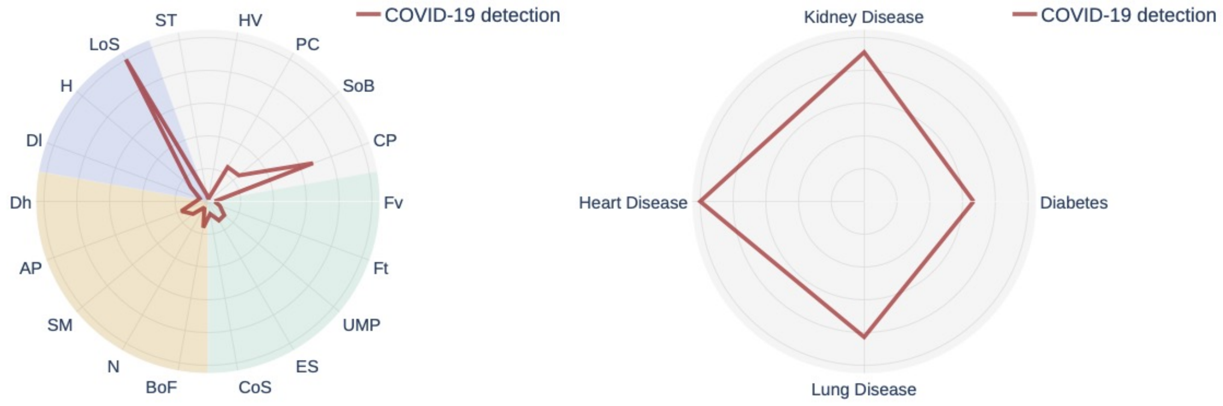

Supplementary Figure 3. Feature relevance to the detection of COVID-19. The left panel presents the symptom relevance by clinical manifestation - gastrointestinal symptoms (yellow section), flu like symptoms (green section), neurological symptoms (blue section) and cardiac and respiratory manifestations (white section). The symptoms considered are encoded as: Fever (Fv), Chest Pain (CP), Shortness of breath (SoB), Persistent Cough (PC), Hoarse Voice (HV), Sore throat (ST), Loss of Smell (LoS), Headache (H), Delirium (Dl), Diarrhoea (Dh), Abdominal Pain (AP), Skipped Meals (SM), Nausea (N), Blisters on Feet (BoF), Chills or Shivers (CoS), Eyes Soreness (ES), Unusual Muscle Pain (UMP), Fatigue (Ft). The right panel shows the relevance of the subjects' comorbidities to the detection of COVID-19. The right panel shows the relevance of the comorbidities to the detection of COVID-19.

Confidence of label prediction

The uncertainty of the prediction of the labels was computed to understand how confident the model is in predicting COVID-19 for age, gender, occupation and BMI subgroups. As a result, for all the correctly predicted labels, we compute the likelihood of the predictions for each of the aforementioned subpopulations. The kernel density function is then obtained from the histogram of the likelihood of the predictions using a Gaussian kernel with bandwidth of 1.5 for all the groups considered. The results are normalised by the number of subjects in each subgroup to correct for the difference of the sample size per group.
When comparing HCW versus NHCW (Supplementary Figure 4), the normalised frequency of the likelihood of the corrected predicted labels shows that in general the model is more certain of the predictions when using the three days of self-reported symptoms information, even though the improvement is not significant from the two days of symptoms. However, the predicted label for HCW subjects is less reliable, even when using longer periods of self-report symptoms.
When used to predict the subject status for the different genders, the model does not show differences in confidence of the predicted labels between the two genders analysed (Supplementary Figure 5). The model presents a confidence between 90-95% for the prediction of the labels for both genders when using either two or three days of information. The model seems not to strongly benefit from the information of the third day of self-reported symptoms to decrease the uncertainty of the estimations.

The uncertainty of the predictions was also analysed by age group. The results, Supplementary Figure 6, suggest that the wider window of symptoms (three days) leads to more certain predictions for all groups of ages, except for patients between 40 and 60 years old. For this group of patients, the results suggest that only two days of information is sufficient to detect with high certainty the early signs of infection. The model shows also more reliable predictions for young to middle-aged patients, from 16 to 60 years old, for whom the average likelihood of the predictions is equal or higher than 90%. The most relevant difference in terms of model certainty regarding the window of information is found for the patients with age above 80 years old, for whom the model requires a bigger window of information in order to have an average certainty above the 90%.

Lastly, we also assessed the certainty of COVID-19 disease prediction for the BMI groups, where four groups are considered including underweight, healthy, overweight and obese patients (Supplementary Figure 7). Despite the general high certainty of the model predictions across groups, for some BMI categories the reported predictions are more uncertain. Specifically, subjects in the extreme BMI groups, namely underweight and obese groups, are predicted as positive SARS-CoV-2 infection with lower certainty. For subjects with low BMI (underweight), the model shows a certainty of only 85-90% for the positive COVID-19 patients. For these patients, the model benefits

of a bigger window of information, showing a higher certainty of the predictions for three days of self-reported symptoms. On the other hand, obese patients are the subjects diagnosed with lower certainty (below 90%). For the obese subjects the additional information seems to increase the noise of the features, which is translated into a lower certainty for the 2 and 3 days of self-reported symptoms. For both healthy and overweight patients, the model has presented high confidence in the predicted labels.

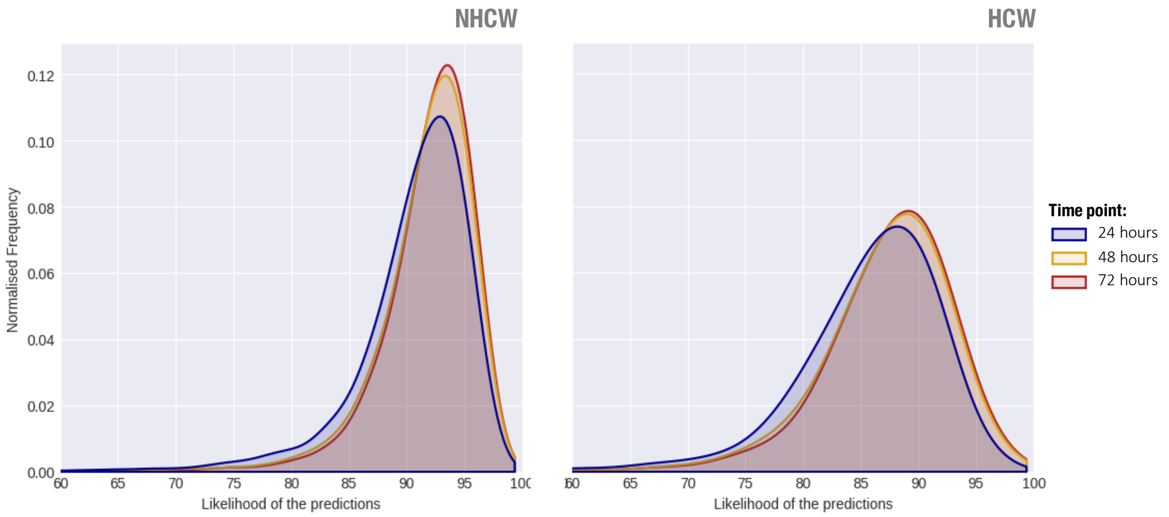

Supplementary Figure 4. Uncertainty estimation of the corrected predicted COVID-19 positive tests by subjects occupation: non-healthcare workers (NHCW) (left panel) *versus* healthcare workers (HCW) (right panel). The different sample size per group is corrected via normalised frequency estimation.

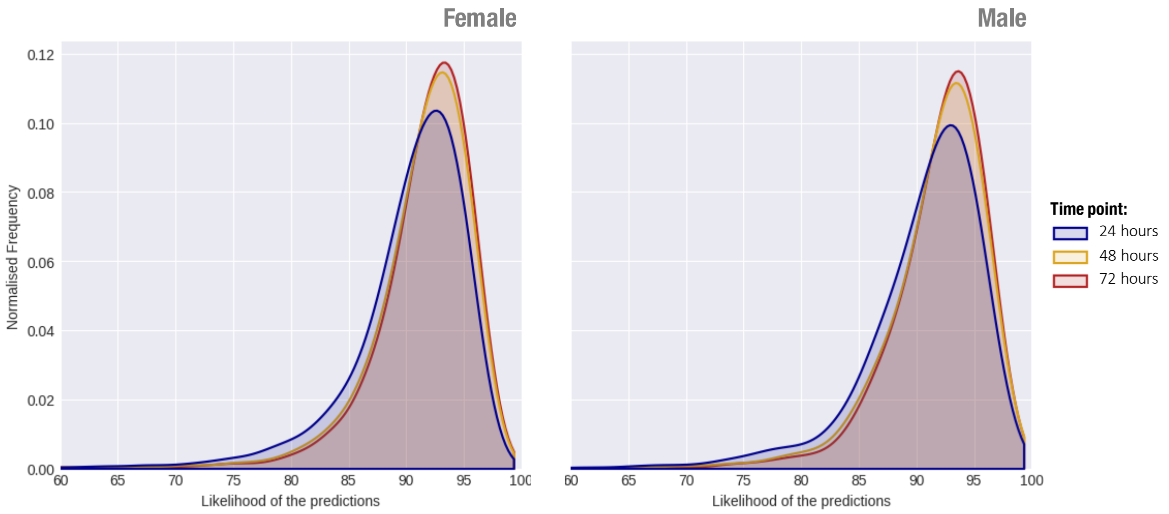

Supplementary Figure 5. Uncertainty estimation of the corrected predicted COVID-19 positive tests per gender group: female (left panel) *versus* male subjects (right panel). The different sample size per group is corrected via normalised frequency estimation.

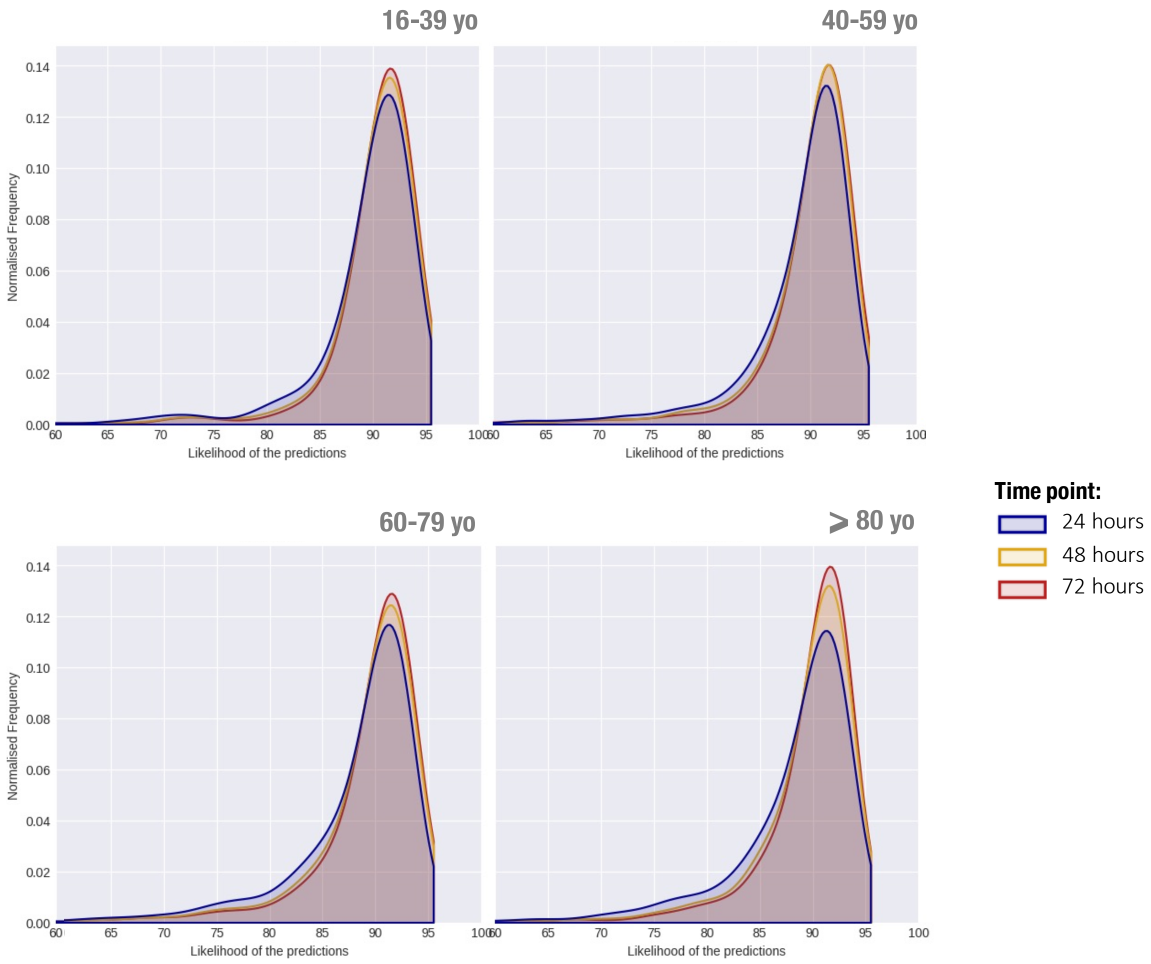

Supplementary Figure 6. Uncertainty estimation of the corrected predicted COVID-19 positive tests stratified by age group. The different sample size per group is corrected via the estimation of the normalised frequency. Yo: years old.

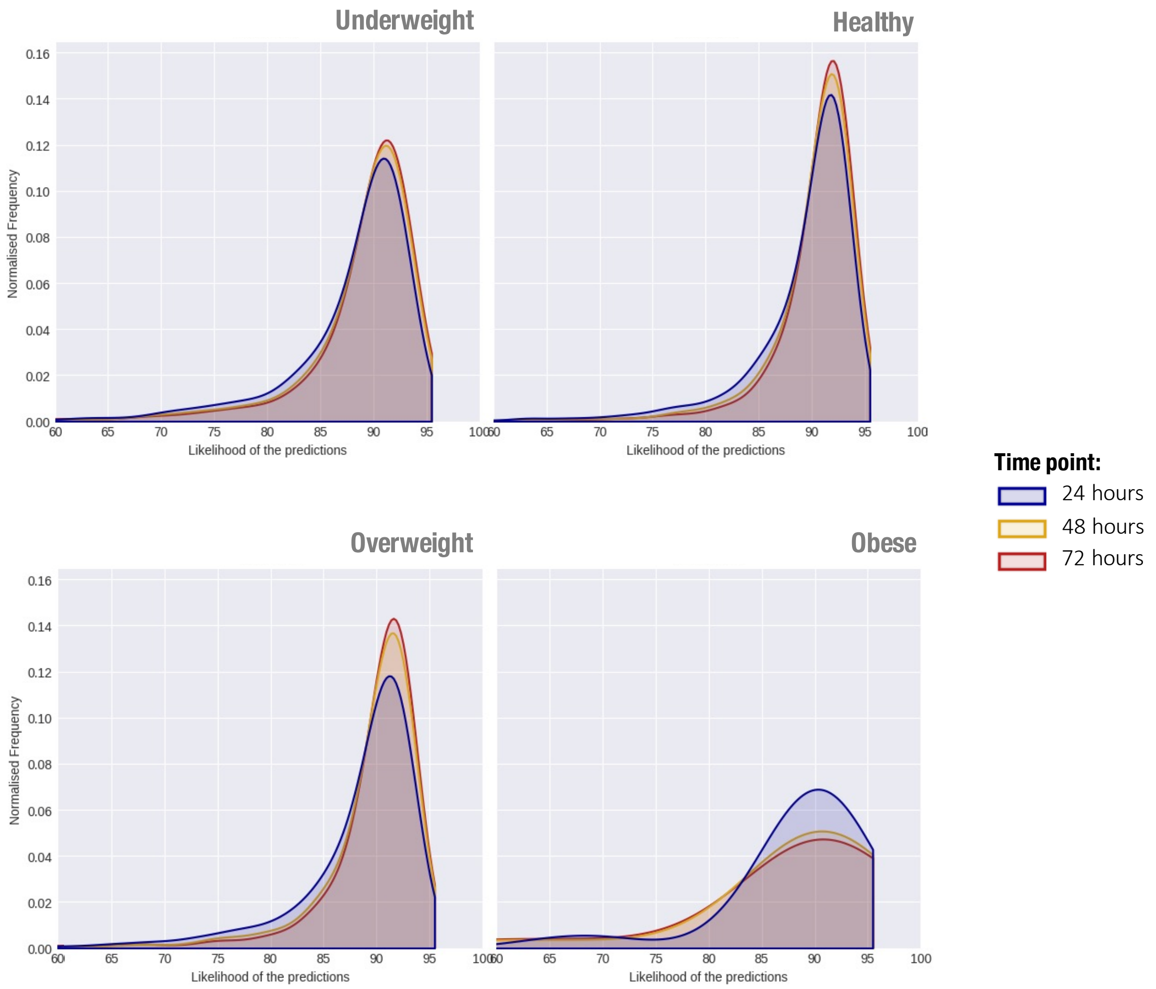

Supplementary Figure 7. Uncertainty estimation of the corrected predicted COVID-19 positive tests stratified by BMI category. The different sample size per group is corrected via the estimation of the normalised frequency.
